# Characterization of *Salmonella* Gallinarum from an outbreak in Raigarh, Chhattisgarh

**DOI:** 10.14202/vetworld.2017.144-148

**Published:** 2017-02-05

**Authors:** Chandrahas Sannat, Anil Patyal, Nidhi Rawat, R. C. Ghosh, D. K. Jolhe, R. K. Shende, S. D. Hirpurkar, Sanjay Shakya

**Affiliations:** 1 Department of Veterinary Microbiology, College of Veterinary Science & Animal Husbandry, Chhattisgarh Kamdhenu Vishwavidyalaya, Anjora, Durg - 491 001, Chhattisgarh, India; 2 Department of Veterinary Public Health, College of Veterinary Science & Animal Husbandry, Chhattisgarh Kamdhenu Vishwavidyalaya, Anjora, Durg - 491 001, Chhattisgarh, India; 3 Department of Veterinary Pathology, College of Veterinary Science & Animal Husbandry, Chhattisgarh Kamdhenu Vishwavidyalaya, Anjora, Durg - 491 001, Chhattisgarh, India

**Keywords:** *bla*_TEM_, O: 9; 12, poultry, *Salmonella* Gallinarum

## Abstract

**Aim:**

The present investigation was conducted to isolate and characterize *Salmonella* Gallinarum from an outbreak of fowl typhoid in layer birds.

**Materials and Methods:**

Clinically ill and dead layer birds from an outbreak were investigated. History, clinical signs, and postmortem lesions were suggestive of fowl typhoid. Postmortem samples including heart blood, intestinal contents, pieces of ovary, and liver were collected and processed immediately for bacterial culture, serotyping and antibiotic sensitivity tests. Isolates were further screened for the presence of extended spectrum beta lactamase (ESBL) (*bla*_TEM_) gene by polymerase chain reaction.

**Results:**

On the basis of cultural, staining and biochemical characteristics; three bacterial isolates were confirmed as S. Gallinarum. On serotyping, somatic antigen O: 9 and 12 with nonflagellated antigen were detected in all three isolates. Isolates were intermediate sensitive to amoxycillin, amoxyclav, gentamicin and ciprofloxacin and resistant to most of the antibiotics including chloramphenicol, ampicillin, ceftazidime, cefexime, cefepime, azithromycin, nalidixin, tetracycline, oxytetracycline, and streptomycin. Two isolates were found to harbor ESBL (*bla*_TEM_) gene.

**Conclusion:**

Beta lactamase producer S. Gallinarum was confirmed as cause of increased mortality in layer birds during present investigation. Existence of multi drug resistant *Salmonella* poses serious threat to poultry industry in Chhattisgarh.

## Introduction

Poultry industry is facing great setbacks due to frequent outbreaks of salmonellosis. Although many developed countries have eradicated these diseases from commercial poultry, it has increased incidence in most developing countries. Fowl typhoid caused by *Salmonella* Enterica subsp. Enterica serovar Gallinarum biovar Gallinarum (*Salmonella* Gallinarum), produces high mortality rates (up to 90%) in birds of all ages, thus causing heavy economic loss [1]. Fowl typhoid has been reported from various parts of India including Kashmir [2], Haryana [3], Kerala [4], and Kolkata [5]; however, reports are lacking from Chhattisgarh state despite its high prevalence, which can be attributed to limited diagnostic facilities under field conditions and under reporting.

Laboratory diagnosis of *Salmonella* relies on selection of suitable sampling procedure combined with a sensitive culture method and further confirmation by biochemical characteristics including IMViC pattern, sugar fermentation, and hydrogen sulfide production [5]. However, control of fowl typhoid is difficult due to lack of proper diagnosis and emergence of antibiotic resistance [6]. The prevalence of drug resistant gene *bla*_TEM_ type extended spectrum beta-lactamases (ESBLs) between *Escherichia coli* and *Klebsiella* has been frequently demonstrated in several countries but it is worth recognizing the emergence of ESBLs in *Salmonella*, which now confers serious clinical problem [7]. The determination of antibiotic susceptibility and multidrug resistance pattern of infectious organisms is therefore necessary to provide a vivid guide for veterinarians to make informed drug choices during the management and treatment of poultry diseases.

The present report sheds light on an outbreak of salmonellosis at Government Poultry Farm in Chhattisgarh (India) describing the disease diagnosis, agent characterization, and antibiotic resistance.

## Materials and Methods

### Ethical approval

No ethical approval was necessary to pursue this research work.

### Sample collection

Increased mortality over a period of 3-week was reported in Giriraj and RIR adult layer birds of age groups 7-8 months at Government Poultry Farm, Raigarh, Chhattisgarh. Birds exhibited acute illness, high temperature, ruffled feather, difficulty in breathing, reduced feed intake, reluctance to move, and watery diarrhea. During postmortem examination, heart blood was collected aseptically from 10 birds in sterilized syringe. On postmortem examination, generalized tissue congestion and dark friable and coppery bronze colored enlarged liver were observed which were suggestive of fowl typhoid. Swab of heart and intestinal contents; tissue pieces of ovary and liver were collected at necropsy of all chickens for bacteriological examination.

### Bacterial isolation

The samples were inoculated immediately in Rappaport Vassiliadis Soya (RVS) peptone broth for selective enrichment of *Salmonella* organisms and incubated at 37°C for 24 h [8]. After selective enrichment, one loopful of each RVS culture was streaked onto on to MacConkey lactose agar (MLA), brilliant green agar (BGA), xylose-lysine-deoxycholate (XLD) agar, and blood agar and incubated at 37°C for 24 h. The nonlactose fermenting colonies of MLA were characterized microscopically using Gram’s-stain.

### Biotyping

Biochemical identification of bacterial isolates was done as described in OIE Manual of Diagnostic Tests and Vaccines for Terrestrial Animals, Volume 1 [8]. Briefly, the tests employed were catalase, oxidase, O/F test, motility test using motility indole urea medium, reactions on triple sugar iron agar (TSI), urease, nitrate reduction, indole, methyl red (MR), Voges Proskauer (VP), citrate utilization, lysine decarboxylase, and sugar fermentation tests (i.e., glucose, sucrose and lactose with an inverted durham tube for acid and gas production, maltose, dulcitol and rhamnose fermentation). All the media used were procured from HiMedia.

### Serotyping

The serotyping of *Salmonella* isolates was performed by National *E. coli* and *Salmonella* Typing Center, Central Research Institute, Kasauli, Himachal Pradesh.

### Antibiotic sensitivity test (ABST)

The ABST was conducted by the disc diffusion method as per Bauer *et al*. [9] in Muller Hinton agar. Antimicrobial inhibition zone diameter was measured and categorized as susceptible, intermediate or resistant. Antibiotic discs (HiMedia) of widely used antimicrobials such as ciprofloxacin (5 µg), cefixime (5 µg), amoxyclav (30 µg), ceftazidime (30 µg), chloramphenicol (30 µg), azithromycin (15 µg), amoxycillin (30 µg), ampicillin (10 µg), cefepime (30 µg), enrofloxacin (10 µg), nalidixin (30 µg), gentamicin (10 µg), tetracycline (30 µg), oxytetracycline (30 µg), and streptomycin (10 µg) were used.

### Detection of multidrug resistant gene

*Salmonella* isolate was further screened for the presence of *bla*_TEM_ gene by polymerase chain reaction (PCR) following the protocol described by Monstein *et al*. [10] with some modifications. For PCR, template DNA incorporated in reactions was prepared by boiling and snap chill method [11]. Purity and concentration of DNA was detected by 0.8% agarose gel electrophoresis and stored at −20°C. Recommended primer set of a *bla*_TEM_ forward primer: 5’-TCGCCGCATACACTATTCTCAGAA TGA -3’ and a *bla*_TEM_ reverse primer: 5’-ACGCTCACCGGCTCCAGATTTAT -3’ were used to obtain a predicted product size of 445 bp (Imperial Life Sciences (P) Limited, Gurgaon, Haryana, India). PCR reactions were performed in a total volume of 25 µl containing ×10 PCR buffer (Tris with 15 mM MgCl_2_), 250 µM of each deoxyribonucleotide triphosphate, 10 pmol of each gene-specific primers, 1 U Taq polymerase and 3 µl of template DNA. PCR amplification was done using thermocycler (Mastercycler, Eppendorf, Germany), and cycles were performed with initial denaturation of 95°C for 10 min; 30 cycles of denaturation at 94°C for 30 s, annealing at 60°C for 30 s, extension at 72°C for 2 min, followed by a final extension step at 72°C for 10 min. After the completion of reaction cycles, the amplified products were electrophoresed on 1.5% agarose gel stained with ethidium bromide (0.5 µg/ml). The images of ethidium bromide stained DNA bands were analyzed under ultraviolet transilluminator (Biometra) and digitized using a gel documentation system (Gel Doc™ XR, Biorad, USA). Reagents used in PCR were procured from Thermo Scientific (USA) and Bangalore Genei (India).

## Results

### Cultural and staining characteristics of bacterial isolates

**Figure-1:**
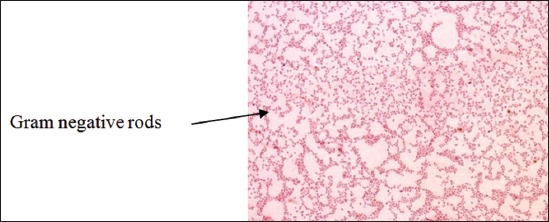
Gram-staining of Salmonella isolates.

**Figure-2:**
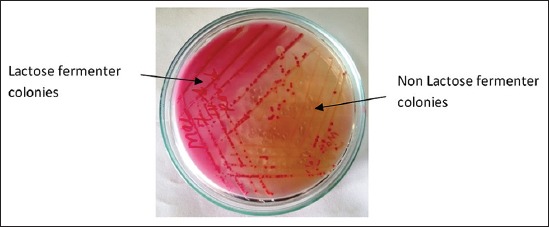
Salmonella colonies on MacConkey agar.

**Figure-3:**
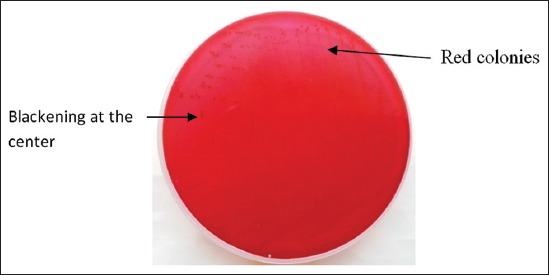
Salmonella colonies on xylose-lysine-deoxycholate agar.

Gram-staining revealed the presence of small rod shape Gram-negative bacteria arranged singly and in pair (Figure-1). RVS culture of heart blood, swab of heart, and intestinal contents showed two types of colony morphology; three isolates showed colorless, translucent, smooth and raised colonies on MLA, indicative of lactose non fermenter organisms and others produced pink color colonies indicative of lactose fermenter organisms (Figure-2). On BGA, nonlactose fermenter isolates produced light pink colony against a rose pink background and was non hemolytic on blood agar. On XLD agar, red colonies were produced initially after 24 h of incubation, which get blackened at center on prolonged incubation (Figure-3). Similarly, on TSI, the reaction occurred slowly with the production of gas and black color colonies. Cultural characteristics of above three isolates were suggestive of *Salmonella* organisms.

### Biochemical profile of bacterial isolates

All three isolates were catalase negative, oxidase positive, fermentative, urease negative, lysine decarboxylase positive, indole negative, MR positive, VP negative, citrate utilization positive, and nonmotile. However, nonmotile organisms were considered to be either *Salmonella* Pullorum or *S*. Gallinarum. During the present investigation, all three isolates fermented glucose, dulcitol, maltose and lysine decarboxylase but not rhamnose, sucrose and lactose which confirmed biovar as *S*. Gallinarum.

### Salmonella serotype

All three *Salmonella* isolates were serotyped as *S*. Gallinarum with somatic antigen O: 9 and 12.

### Antibiogram of S. Gallinarum

*Salmonella* Gallinarum isolates were intermediate sensitive to amoxyclav, amoxycillin, gentamicin, ciprofloxacin and enrofloxacin however resistant to chloramphenicol, ampicillin, ceftazidime, cefexime, cefepime, azithromycin, nalidixin, tetracycline, oxytetracycline and streptomycin.

### ESBL bla_TEM_ gene

**Figure-4:**
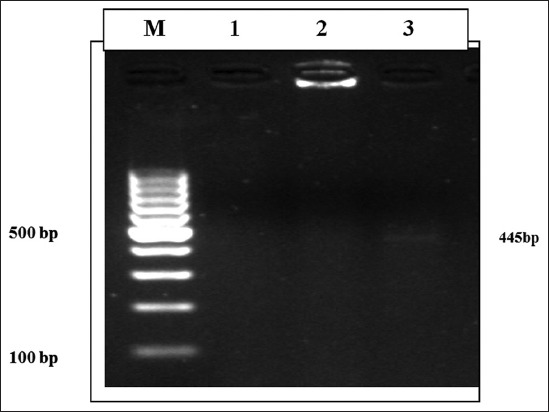
Agarose gel electrophoresis showing amplified polymerase chain reaction product of bla_TEM_ gene. Lane M: 100 bp DNA ladder, Lane 1, 2: Salmonella isolates with no amplicon, Lane 3: Salmonella Gallinarum isolate with bla_TEM_ positive amplicon (445 bp).

Two isolates of *S*. Gallinarum were found to harbor the drug resistant *bla*_TEM_ gene having amplicon size of 445 bp (Figure-4).

## Discussion

Salmonellosis is becoming serious problem in developing country like India since control measures are not efficient and also the climatic conditions favor the environmental spread of these organisms [12] which might lead to increased incidence of salmonellosis. Likewise present study, Kumari *et al*. [1], Ambily and Mini [4], and Dey *et al*. [5] also recorded increased mortality due to fowl typhoid. Clinical signs and gross pathology reported in present investigation corresponds with the findings of Kumari *et al*. [1] who also noticed swollen and congested liver with bronze discoloration in cases of fowl typhoid.

Since the isolation and correct identification of *Salmonella* are very crucial for the characterization, the colonies having typical cultural characteristics were selected as presumptive for *Salmonella* serovars. Cultural morphology on XLD, MLA and BGA was in accordance with the findings of Park *et al*. [13] and Ferdous *et al*. [14]. Differentiation of two nonmotile biovars, viz., *S*. Gallinarum and *S*. Pullorum done by specific sugar fermentation tests concurs with the report of Rahman *et al*. [15]. Biochemical reactions and antigenic formula reported during present investigation were classical findings of *S*. Gallinarum [5,16].

The widespread and irrational use of antimicrobial drugs in poultry production has contributed to the emergence and maintenance of resistance genes particularly ESBLs genes, which poses serious threat not only to poultry industry but also of public health concern [17]. Similar to present findings, multidrug resistant *Salmonella* serovar were reported by Zafer Ata *et al*. [18] and Andoh *et al*. [19], whereas limited drug resistance in *S*. Gallinarum was observed by Dey *et al*. [5]. Likewise present study, Kumari *et al*. [1] reported *S*. Gallinarum isolates sensitive to ciprofloxacin, enrofloxacin and gentamicin, amoxicillin, amoxyclav, and resistant to nalidixic acid. *Salmonella*. Gallinarum isolates sensitive to ciprofloxacin and resistant to chloramphenicol were reported by Parvej *et al*. [20] and Ferdous *et al*. [14], respectively, which also concur our present finding. In contrast, Kumari *et al*. [1] reported reemergence of chloramphenicol sensitivity, and Filho *et al*. [6] reported reduced susceptibility of isolates to enrofloxacin, ciprofloxacin, norfloxacin, and ofloxacin. Emergence of ESBLs genes in salmonellae poses serious problem in management and treatment of salmonellosis in poultry [21,22]. In hormony with the present findings, earlier study also reported, *bla*_TEM_ positive salmonellae isolates of poultry resistant to ampicillin [23]; amoxyclav, ampicillin and cefalothin [24] and nalidixic acid, chloramphenicol, tetracycline and trimethoprim [22].

## Conclusion

ESBL producer *S*. Gallinarum was reported as the cause of increase mortality in layer birds at poultry farm of Chhattisgarh. Emergence of multidrug resistant *Salmonella* with ESBLs activity during this study pretense a serious threat for future treatment options in poultry industry in Chhattisgarh.

## Authors’ Contributions

CS designed the experiment under supervision of SDH. Postmortem examination was made by RCG and DKJ. Media preparation, sample collection and bacteriological analysis were performed by CS, NR and RKS. Molecular work was performed by AP and SS. All authors participated in draft and revision of the manuscript. All authors read and approved the final manuscript.
